# Defective hematopoietic differentiation of immune aplastic anemia patient-derived iPSCs

**DOI:** 10.1038/s41419-022-04850-5

**Published:** 2022-04-28

**Authors:** Maria Florencia Tellechea, Flávia S. Donaires, Vinícius S. de Carvalho, Bárbara A. Santana, Fernanda B. da Silva, Raissa S. Tristão, Lílian F. Moreira, Aline F. de Souza, Yordanka M. Armenteros, Lygia V. Pereira, Rodrigo T. Calado

**Affiliations:** 1 Department of Medical Imaging, Hematology, and Oncology, Ribeirão Preto Medical School, University of São Paulo, Ribeirão Preto, São Paulo, Brazil; 2 Department of Veterinary Medicine, Faculty of Animal Science and Food Engineering, University of São Paulo, Pirassununga, São Paulo, Brazil; 3 Department of Genetics and Evolutionary Biology, Biosciences Institute, University of São Paulo, São Paulo, Brazil

**Keywords:** Anaemia, Induced pluripotent stem cells

## Abstract

In acquired immune aplastic anemia (AA), pathogenic cytotoxic Th1 cells are activated and expanded, driving an immune response against the hematopoietic stem and progenitor cells (HSPCs) that provokes cell depletion and causes bone marrow failure. However, additional HSPC defects may contribute to hematopoietic failure, reflecting on disease outcomes and response to immunosuppression. Here we derived induced pluripotent stem cells (iPSCs) from peripheral blood (PB) erythroblasts obtained from patients diagnosed with immune AA using non-integrating plasmids to model the disease. Erythroblasts were harvested after hematologic response to immunosuppression was achieved. Patients were screened for germline pathogenic variants in bone marrow failure-related genes and no variant was identified. Reprogramming was equally successful for erythroblasts collected from the three immune AA patients and the three healthy subjects. However, the hematopoietic differentiation potential of AA-iPSCs was significantly reduced both quantitatively and qualitatively as compared to healthy-iPSCs, reliably recapitulating disease: differentiation appeared to be more severely affected in cells from the two patients with partial response as compared to the one patient with complete response. Telomere elongation and the telomerase machinery were preserved during reprogramming and differentiation in all AA-iPSCs. Our results indicate that iPSCs are a reliable platform to model immune AA and recapitulate clinical phenotypes. We propose that the immune attack may cause specific epigenetic changes in the HSPCs that limit adequate proliferation and differentiation.

## Introduction

Aplastic anemia (AA) is a hematopoietic stem and progenitor cell (HSPC) disorder clinically characterized by a hypoplastic bone marrow and low peripheral blood (PB) counts [1]. Pancytopenia is life-threatening due to hemorrhage and infection complications. AA is the result of HSPC failure, which may be due to inherited genetic defects or an acquired mechanism in which HSPCs are the target of an immune process [1]. Among the inherited cases, germline pathogenic variants in the DNA repair machinery and telomere-related genes are common [2]. In acquired cases, no germline genetic defect in HSPCs is apparent, and marrow failure results from an immune-mediated HSPC depletion promoted by the expansion of cytotoxic Th1 cells targeting CD34^+^ cells, inducing apoptosis via the Fas-FasL pathway [3].

Severe immune AA cases may be cured with a sibling-matched allogeneic hematopoietic stem cell transplantation (HSCT). However, most patients lack a suitable donor or are not eligible for transplant, and intensive immunosuppressive therapy (IST) with anti-thymocyte globulin (ATG) and cyclosporine associated with eltrombopag is preferred [4]. IST with eltrombopag achieves hematologic response in up to 90% of cases, but relapse is a major concern, occurring in one third of responders [4]. As eltrombopag, a thrombopoietin mimetic and a stem cell stimulator, significantly improves response rates, treatment failure may be attributed to the scarce quantity of the remaining HSPCs at diagnosis [1, 5]. Alternatively, the lack of response and frequent relapse may be due to HSPC defects or to lesions inflicted to the HSPC compartment by the immune attack.

Human induced pluripotent stem cells (iPSCs) [6] are a source of patient-specific hematopoietic cells to model diseases and a potential source of hematopoietic tissue for cell therapies. Hematopoietic differentiation from human iPSCs can model bone marrow failure syndromes in vitro [7]. For instance, we have recently demonstrated that the pathogenic *DKC1* p.A353V variant, which causes dyskeratosis congenita, provokes severe telomere attrition during reprogramming and hematopoietic differentiation skewing toward primitive hematopoietic progenitors [8].

Immune AA also have been modeled by iPSCs. Espinoza et al. derived iPSCs from AA patients’ cells lacking HLA class I alleles due to 6p loss of heterozygosity (6pLOH) and showed that CD34^+^ cells derived from HLA-deficient iPSCs escaped the immune attack by cytotoxic T cells [9]. Another study derived iPSCs from severe AA patients and observed defective iPSCs telomere elongation and impaired hematopoietic differentiation [10]. These deficiencies were not rescued by eltrombopag and was attributed to cryptic genetic defects in AA patients’ cells, as deleterious variants in telomere-associated genes (*RPA2*, *NCL*, *POLD3*, *TEP1*, *YLMP1*, *PIF1*, and *ERCC4*) were identified by exome sequencing in patient’s fibroblasts [10].

Here, we derived iPSCs from patients diagnosed with immune AA who responded to IST, had normal-for-age telomeres, and carried no pathogenic variants in inherited AA-related genes by next-generation sequencing. We investigated their hematopoietic differentiation potential and telomere functionality.

## Results

### Immune AA patients’ erythroblasts reprogramming into iPSCs

We tested the reprogramming capacity of somatic cells from three patients diagnosed with immune AA and three healthy controls (Table 1). All patients responded to immunosuppression with ATG and cyclosporine according to standard criteria [11] and PB leukocyte samples were collected for reprogramming after hematologic recovery was achieved and sustained. Two patients (AA-1 and AA-2, who achieved partial remission) had detectable (>1%) glycosylphosphatidylinositol (GPI)-negative clones in both neutrophils (35% and 6%) and erythrocytes (6% and 2%), further supporting an immune-mediated pathophysiology. Patients had negative DEB test, normal-for-age telomere lengths and did not carry any identifiable pathogenic or likely pathogenic germline variant in genes known to be associated with inherited bone marrow failure syndromes (Supplementary Table 1) [1, 12].

**Table 1:** Clinical characteristics of patients and healthy controls.

Individual (Gender, age)	Diagnosis	BM cellularity	PNH clone (%Neutrophils/Erythrocytes)	Treatment response	Telomere length (kb)^a^
**AA-1**	Moderate AA	45%	34.8/5.5	rATG+CSA	10.7
(F, 29)				Partial	
**AA-2**	Severe AA	<10%	6/2	rATG+CSA	7.7
(M, 17)				Partial	
**AA-3**	Severe AA	<25%	0.02/0.05	hATG+CSA	6.5
(M, 51)				Complete	
**Ctrl-1**	Unaffected	N/A	N/A	N/A	8.6
(F, 29)					
**Ctrl-2**	Unaffected	N/A	N/A	N/A	N/A
(F, 66)					
**Ctrl-3**	Unaffected	N/A	N/A	N/A	N/A
(M, 43)					

*BM* bone marrow, *kb* kilobases, *N/A* not available, *r/hATG* rabbit/horse antithymocyte globulin, *CSA* Cyclosporine A, *PNH* Paroxysmal nocturnal hemoglobinuria.

^a^Telomere length measured by flow-FISH.

**Fig. 1:**
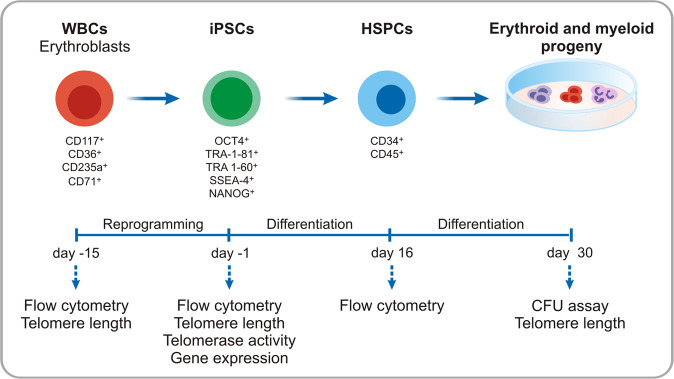
Schematic representation of experiments performed for modeling AA in vitro. WBCs, White Blood Cells; iPSCs, induced Pluripotent Stem Cells; HSPCs, Hematopoietic Stem and Progenitor Cells; CFU, Colony-Forming Unit.

**Fig. 2:**
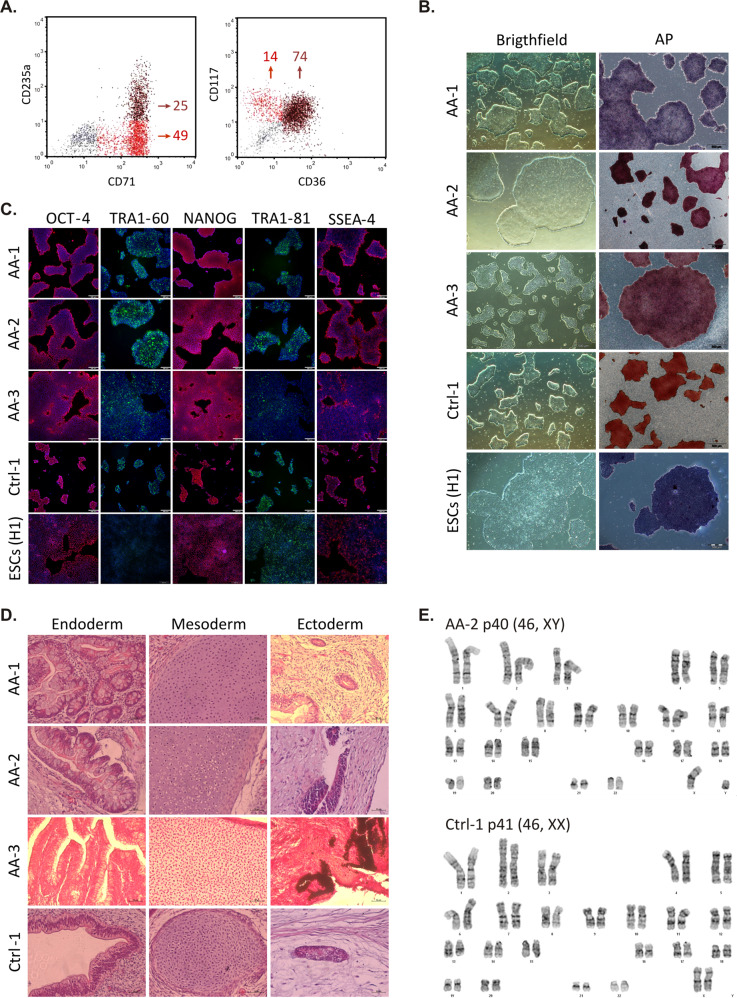
AA and healthy-iPSC lines display hallmarks of pluripotency similar to ESCs (H1). **A** Immunophenotype of erythroblasts from Ctrl-1 after 10 days in culture. Percentages of each population in two-color dot plots are indicated. The majority of cells resemble erythroblasts co-expressing CD71^high^ (transferrin receptor) in red dot plots and CD235a (glycophorin A) in dark red dot plots. Expanded erythroblasts also expressed CD117 (earliest pro-erythroblast stage marker, in red dot plots) and the conventional erythroid-associated marker CD36 (dark red dot plots). Red and dark red dot plots indicate immature and mature stages of the cells, respectively. **B** Images of AA and healthy-iPSCs showing typical morphology and staining for alkaline phosphatase (AP) similar to the ESC line (H1). Scale bars, 500 μm. **C** Images of AA and healthy-iPSC colonies staining for pluripotency markers by immunocytochemistry. DAPI staining is shown in blue. Scale bars, 200 μm. **D** In vivo pluripotency test of AA and healthy-iPSCs assessed by teratoma formation assay. The presence of tissue derived from the embryonic germ layers endoderm, mesoderm and ectoderm are shown in representative images of H&E stained sections. Scale bars, 50 μm. **E** Representative karyograms of iPSCs derived from patient AA-2 and Ctrl-1 at passage 40 (p40), showing normal karyotypes (2*n* = 46). Ctrl-1-iPSCs characterization is representative of the healthy iPSC controls.

To derive iPSCs from patients and controls, PB samples were collected, mononuclear cells separated and cultured with specific cytokines for erythroblast expansion (Fig. 1). After 10 days in culture, more than 70% of expanded cells expressed erythroid-associated markers: CD117, CD36, CD235a, and CD71 (Fig. 2A), as previously reported [13]. The AA and healthy expanded erythroblasts were successfully reprogrammed to iPSCs using non-integrating plasmids [14] and one or two iPSC clones from each subject was selected for the subsequent experiments. The reprogramming efficiency was 2.1 × 10^−6^% ± 0.7 × 10^−6^% (mean ± standard deviation) in AA samples and 5.2 × 10^−6^% ± 1 × 10^−6^% (mean ± standard deviation) in healthy control samples. The reprogrammed AA and healthy cells displayed typical characteristics of embryonic stem cells (ESCs): morphology, positive staining for alkaline phosphatase, and expression of the pluripotency markers OCT4, TRA-1-60, NANOG, TRA-1-81, and SSEA-4 (Fig. 2B and C).

The in vivo differentiation potential of each iPSC clone also was evaluated. The AA and healthy iPSCs were able to form teratomas in NSG mice, containing cells from the three germ layers resembling glands and gut cells (endodermal origin), chondrocytes (mesodermal origin), and pigmented retinal epithelium or neural structures (ectodermal origin), Fig. 2D.

In order to screen for potential episomal plasmid DNA integration in the iPSCs, we verified the *EBNA1* gene fragment amplification that is common to the eight reprogramming vectors used in this study. After 14 passages, *EBNA1* was undetectable in all patient and control iPSC clones (Supplementary Fig. 1) [13]. Cytogenetic analysis by G-banding showed normal karyotypes in all the iPSC clones studied, suggesting that the reprogramming procedure and culture conditions did not cause detectable genomic abnormality in the healthy and AA-iPSCs (Fig. 2E).

These results demonstrate that immune AA erythroblasts are successfully reprogrammed to a pluripotent state similar to ESCs, without evidence of genomic instability or any remaining exogenous DNA.

### Differentiation into hematopoietic progenitors is impaired for immune AA-iPSCs

**Fig. 3:**
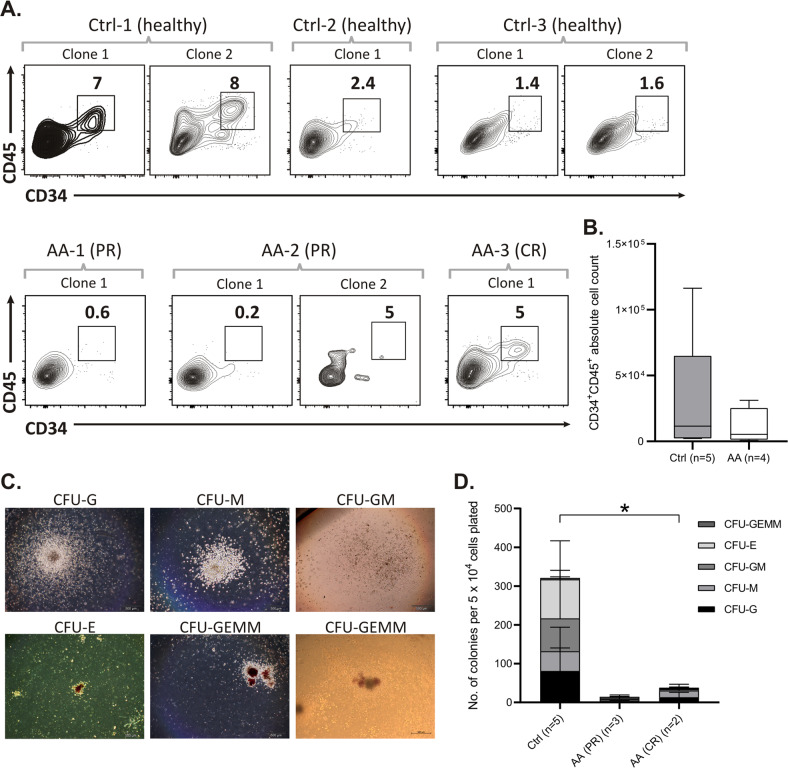
AA and healthy-iPSCs differentiation into hematopoietic progenitors. **A** Representative images of flow cytometry analysis in percentages of CD34 and CD45 co-expression in healthy and AA cells at day 16 of hematopoietic differentiation. Ctrl-1 (clones 1 and 2), Ctrl-2 (clone 1) and Ctrl-3 (clones 1 and 2) represent healthy subjects; Patients samples AA-1 (clone 1) and AA-2 (clones 1 and 2) are partially responders (PR) to immunosuppression and AA-3 (clone 1) had a complete response (CR) to immunosuppression. **B** Box-plot showing the absolute cell count from the CD34^+^CD45^+^ subset derived from Ctrl (3 subjects, 5 clones) and AA (3 subjects, 4 clones) iPSCs. The center line denotes the median value (50th percentile), while the box contains the 25th to 75th percentiles of dataset. The black whiskers indicate the minimum and maximum values. **C** Morphology of representative myeloid and erythroid colonies from a colony forming unit (CFU) assay: CFU-G (granulocytes) CFU-M (macrophages), CFU-GM (granulocytes and macrophages), CFU-E (erythrocytes) and, CFU-GEMM (granulocytes, erythrocytes, monocytes, megakaryocytes). Scale bars, 500 μm. **D** Qualitative and quantitative analysis of hematopoietic colonies derived from healthy (3 subjects, 5 clones) and AA-iPSC (3 subjects, 5 clones). Data is presented as mean of experimental triplicates ± SD.

The derivation of HSPCs from patient-specific iPSCs aids the study of tissue-depleted disorders, such as AA [8, 15]. We selected iPSCs from three AA patients (total of five clones) and three healthy donors (total of five clones) to differentiate into hematopoietic tissue using the embryoid body system in serum-free medium supplemented with cytokines (Fig. 1). After 16 days in culture, the absolute number of hematopoietic-committed cells (CD34^+^CD45^+^) was on average 2.7 times lower in AA-iPSCs than in healthy iPSCs (Fig. 3A, B). There was 97% concordance between the CD43 and CD45 expression within the CD34^+^ population (Supplementary Fig. 2). Of note, iPSC hematopoietic differentiation appeared to recapitulate the clinical phenotype, as iPSCs derived from the patient who achieved complete response (AA-3) displayed better hematopoietic differentiation than those derived from patients who were partial responders (AA-1 and AA-2), whose hematopoietic differentiation was minimal.

The same pattern was observed in the colony forming unit (CFU) assays, which are useful to functionally evaluate the hematopoietic progenitors. The colonies were classified into CFU-G, CFU-M, CFU-GM, and CFU-E according to their morphology (Fig. 3C). The total number of colonies was significantly lower in the three AA-iPSC differentiated samples in comparison to the healthy iPSC differentiated cells (Ctrl, 73 ± 65 vs. AA, 5 ± 4; media ± standard deviation; *p* = 0.03; Fig. 3D; Supplementary Fig. 3). Again, the AA-3-iPSCs (from the patient who achieved complete response) produced a higher number of colonies than the iPSCs derived from the two patients who achieved partial remission (AA-1 and AA-2). Altogether, the results suggest that our hematopoietic differentiation system accurately reproduced the AA phenotype in vitro.

### The telomere repair machinery is functional and preserved in immune AA iPSCs

**Fig. 4:**
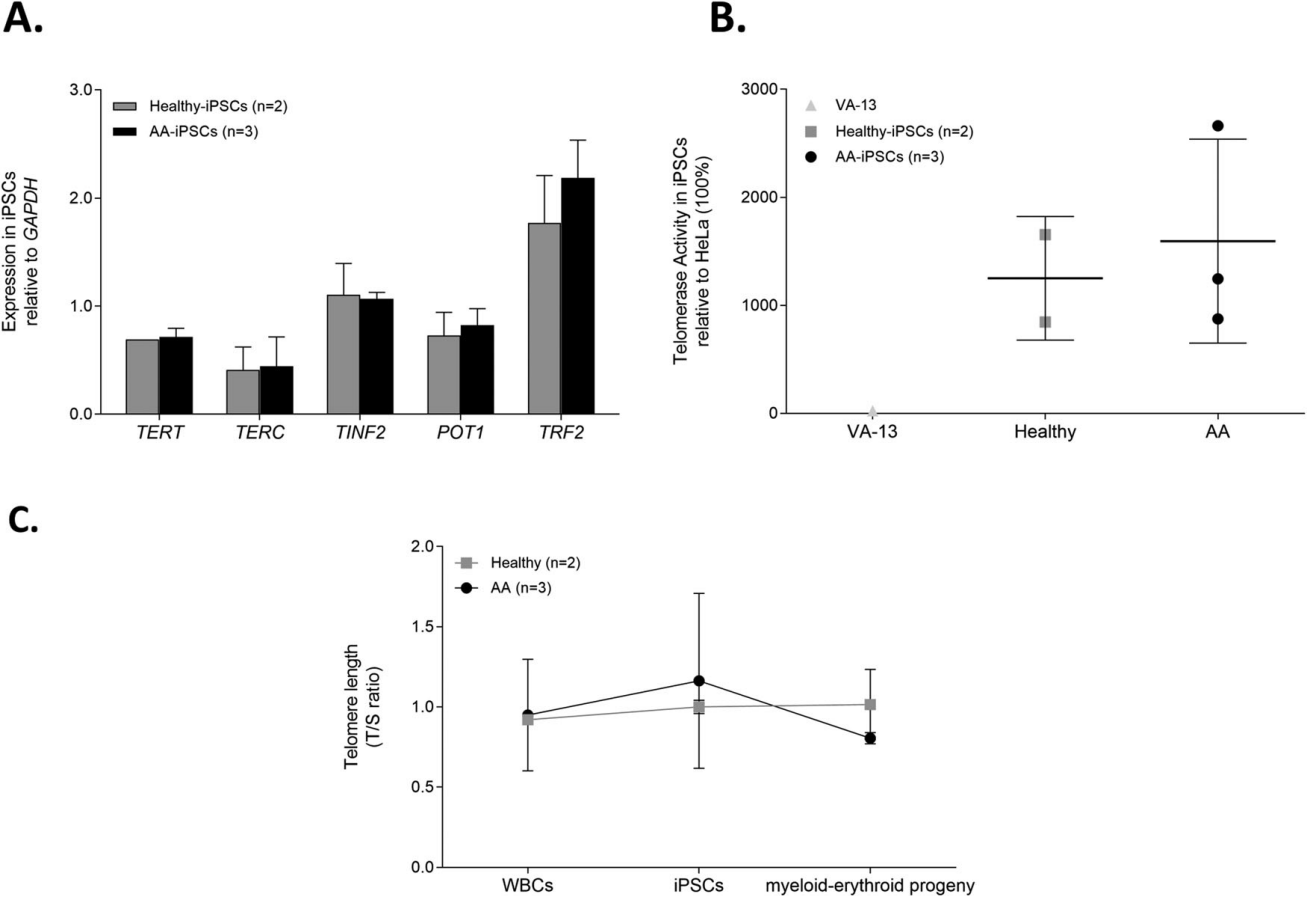
Telomere biology of healthy and AA cells. **A** Gene expression analyses of *TERT*, *TERC*, *TINF2*, *POT1* and *TRF2* from AA (*n* = 3) and healthy-iPSCs (*n* = 2). The genes are listed on the x-axis, and the mean fold change of expression is shown on the *y*-axis. H1 lineage was used as the reference group. Fold changes were calculated using the ddCT method, in which fold change data are represented as 2^−ddCT^. Bars represent mean ± SD. **B** Telomerase activity, relative to HeLa, was detected in AA (*n* = 3) and healthy-iPSCs (n = 2). The ESC line H1 was considered as positive control for telomerase activity and the telomerase-deficient VA-13 cell line as negative control. **C** Telomere dynamics in healthy and AA cells. Telomeres were measured in WBCs, iPSCs and cells derived from CFU assay by qPCR and presented by T/S ratio. The telomeres in AA cells behaved similar than the healthy group during the reprogramming and differentiation process. Data is presented as mean of independent experimental and biological replicates ± SD.

To adequately model AA, we investigated potential telomere and telomere repair abnormalities in AA-iPSCs. First, the patients were screened for germline pathogenic variants in 158 bone marrow failure-related genes (Supplementary Table 1), but no variant was identified in any of the three AA patients. Next, we analyzed the gene expression, telomerase activity, and the telomere length of iPSCs derived from AA patients and healthy controls. We found that the mRNA levels of telomere-related genes (*TERT*, *TERC, TINF2*, *POT1*, and *TRF2*) were comparable in AA patient and healthy iPSCs (Fig. 4A). We also observed that telomerase was equally active in AA-iPSCs and healthy-iPSCs (Fig. 4B).

We finally assessed the telomere dynamics from reprogramming to differentiation by analyzing samples from somatic, pluripotent, and blood-differentiated cells of both AA patients and healthy donors. Before reprogramming, the telomere length of the patients’ leukocytes was in the normal-for age range. After reprogramming, telomere length was equally maintained in both AA and healthy iPSCs. We then measured telomeres after hematopoietic differentiation (30 days of differentiation) and found that telomere lengths were comparably maintained in both groups (Fig. 4C). In aggregate, these findings support that the telomere repair machinery was preserved during reprogramming and differentiation of AA-iPSCs, further recapitulating the clinical phenotype.

## Discussion

In the present study, we successfully reprogrammed PB erythroblasts of patients with immune AA into pluripotent state, and the resulting iPSCs reliably recapitulated disease phenotype. Hematopoietic differentiation of AA-iPSCs was impaired both quantitatively and qualitatively; it appeared to be more severely diminished in iPSC clones derived from patients with partial response to immunosuppression and less impacted in the iPSC clone derived from the patient who achieved complete remission. Reprogramming fully activated the telomerase machinery, elongated telomeres, and appropriately maintained their lengths during hematopoietic differentiation. These in vitro findings reiterate the HSPC function observed in vivo in patients after hematologic response, supporting the iPSC system as a reliable disease model for immune AA.

Acquired immune AA results from an immune destruction of healthy HSPCs mediated by cytotoxic T cells [1]. That the dysregulated activation of effector T cells cause the failure of otherwise healthy HSPCs is illustrated by the development of AA after the use of checkpoint inhibitors in cancer treatment [16]. Additionally, pathogenic variants in genes involved in immune regulation, such as *CTLA4*, were also clinically present in apparently “acquired” immune AA [17]. However, it is possible that HSPCs may be defective prior to the immune process and the “defect” per se may serve as a trigger to the immune system. Melguizo-Sanchis et al. (2018) derived iPSCs from skin fibroblasts obtained from three patients diagnosed with severe AA (SAA) and observed deficient hematopoietic differentiation and an inability to maintain their telomeres, suggesting a genetic defect underlying the “acquired” disease. Exome sequencing of their skin fibroblasts revealed pathogenic variants in telomere-associated genes (*RPA2*, *NCL*, *POLD3*, *TEP1*, *YLMP1*, *PIF1*, and *ERCC4*), further suggesting a genetic predisposition. However, the three patients selected in that study were not adequately screened for an acquired immune mechanism and may not represent immune AA. Only one patient responded to immunosuppression, specific drugs were not detailed, and response intensity and criteria were not specified. The other two patients received unrelated matched hematopoietic stem cell transplant. One patient was 10 years old and had other phenotypes, suggesting an inherited bone marrow failure syndrome.

Here, we carefully selected three immune AA cases who responded to ATG and cyclosporine according to standard criteria [11]. Patients were previously screened for germline pathogenic variants (Supplementary Table 1) and no mutation was identified. Two patients had overt GPI-negative clones. In agreement with the previous study, we also found defective hematopoietic differentiation of AA-patient-derived iPSCs. However, in contrast with Melguizo-Sanchis et al. [10], telomere repair machinery and telomere length were fully preserved. Our findings suggest that the hypothesis by Melguizo-Sanchis et al. [10] of a cryptic genetic defect is less likely, as telomere lengths were in the normal range in PB leukocytes and maintained during reprogramming. Very short telomeres are characteristic of telomere diseases, but other inherited bone marrow failure syndromes also invariably have short-for-age telomeres [18]. We have recently developed a genome-based machine-learning predictor of inherited AA and telomere length emerged as the most important variable for the model’s predictive accuracy [19].

In aggregate, our findings support an alternative hypothesis that the immune attack may cause deleterious long-lasting epigenetic changes in target HSPCs, limiting their proliferation and differentiation. The transcription factors involved in reprogramming (OCT4, SOX2, KLF4, and MYC) bind to the chromatin and induce remodeling by replacing tissue-specific epigenetic patterns with pluripotency-associated modifications. Epigenetic modifications regulate gene transcription and genomic stability governing cellular mechanisms of proliferation and differentiation. However, iPSCs still retain an epigenetic memory of their tissue of origin that favors their differentiation along lineages related to the donor cell [20]. Here, we used AA-derived erythroblasts (hematopoietic origin) obtained after response to immunosuppression to induce pluripotency and achieved successful iPSC reprogramming efficiency. However, in spite of the blood origin, AA-iPSCs had defective hematopoietic differentiation that recapitulated patients’ phenotypes: those with partial response tended to show lower hematopoietic differentiation capacity than the one patient with complete response in independent experiments and in different clones. This blockade was observed both quantitatively (by flow cytometry) and qualitatively (in colony-formation assays). We speculate that the immune attack against HPSCs in immune AA provoked specific epigenetic changes that were retained by AA-iPSCs and affected their hematopoietic differentiation potential.

The potential epigenetic signature inflicted by the immune-mediated attack may explain some characteristics of the hematologic response to immunosuppression. First, it may elucidate why most patients display partial remission once the pathogenic T-cells are removed by ATG and cyclosporine, as the epigenetic events in HSPCs may limit hematopoiesis. Second, it may explain why eltrombopag added to immunosuppression results in higher complete and overall response rates [4]. Eltrombopag preferentially expands hematopoietic multipotent progenitors [21], improving hematopoiesis, but the exact mechanisms are not understood. It is possible that eltrombopag, a thrombopoietin receptor agonist, may impact on epigenetic markers in HSPCs.

Our study has limitations. First, we were unable to obtain skin fibroblasts from AA patients for reprogramming to compare their hematopoietic differentiation capacity. Second, the number of patients studied is limited (five iPSC clones derived from three patients). However, it is comparable to other previous studies and limited by the laborious and expensive reprogramming and differentiation experiments. Finally, we did not analyze specific epigenetic changes in our AA-iPSCs. Epigenetic signatures were not pursued due to the lack of reprogrammed skin fibroblasts for adequate comparison.

In summary, our study shows that iPSCs are a reliable platform to model immune AA and adequately recapitulate the clinical phenotypes. Based on our findings, we propose that the immune attack causes specific epigenetic changes in the HSPCs that are persistent and that warrant further investigation.

## Methods

### Patients and controls

For this study we recruited three patients diagnosed with aplastic anemia [22, 23], two males with severe AA and one female with moderate AA; average age, 32.3 years old (Table 1). Patients received immunosuppressive treatment with h/rATG+CSA reaching partial or complete response. The patients had normal telomere length (TL) for their ages in white blood cells (WBCs), and no pathogenic variants in 158 bone marrow syndromes related-genes (Supplementary Table 1). We also included control samples from three healthy donors; average age, 46 years old (Table 1). PB samples were collected at the University Hospital, Ribeirão Preto Medical School, University of São Paulo, after approval by the local ethics committee (HCRP 6212/2014), following written informed consent of patients.

### Erythroblasts immunophenotyping

Expanded erythroblasts were labeled with anti-CD71 (FITC), 235a (PE), 36 (FITC) and 117 (PE), (BD Biosciences, East Rutherford, New Jersey, U.S.). Cells were acquired in BD FACSCalibur™ flow cytometer and viable 7-AAD (BD Biosciences) negative cells were analyzed in FlowJo V10 software (BD Biosciences).

### Derivation of iPSCs from AA patients and healthy donors

iPSCs from AA patients and healthy donors were derived as previously described, with modifications [14]. Briefly, mononuclear cells (MNCs) from PB were cultured for 10 days in erythroblasts stimulating medium, STEM SPAN (STEMCELL Technologies, Vancouver, Canada) supplemented with 40 ng/mL IGF-1, SCF, 10 ng/mL IL-3 (R&D Systems, Mineapolis, Minnesota, U.S.), 2 U/mL EPO (Blau, São Paulo, Brazil) and 1 μM/mL dexamethasone (Sigma, Saint Louis, Missouri, U.S.). Erythroblasts were transfected with episomal plasmids pCE-hOCT3/4 (*POU5F1*), pCE-hSK (*SOX2* and *KLF4*), pCE-hUL (*LIN28* and *L-MYC*), pCE-mp53DD (*Trp53*) and pCXB-EBNA1 (*EBNA-1*), (Addgene, Watertown, Massachusetts, U.S.) [24]. Nucleoporation was performed using the Human CD34^+^ Cell Nucleofector^TM^ kit and the Amaxa Nucleofector 2b (Lonza, Basel, Switzerland). Three days after transfection, cells were seeded into irradiated murine embryonic fibroblasts (MEFs; GlobalStem, Gaithersburg, Maryland, U.S.) supplemented with 0.25 mM sodium butyrate (Sigma-Aldrich) until colonies emergence. Cells were cultured at 37 °C, 5% CO_2_. The healthy iPSC line, Ctrl-2, was gently ceded by the National Laboratory of Embryonic Stem-Cells (LaNCE), Institute of Biosciences, University of São Paulo, SP, Brazil. This iPSC line was generated using different reprogramming plasmids [25]. The H1 embryonic stem cell line (WiCell, Madison, Wisconsin, U.S.) was used as a positive control for characterization.

### Alkaline phosphatase detection assay

Alkaline phosphatase was assessed in iPSCs using Alkaline Phosphatase Detection kit (Sigma-Aldrich) following manufacturer’s instructions. Briefly, colonies were fixed and incubated in an alkaline solution. Finally, cells were stained with Hematoxilin n°3 of Gill and images were captured with an Olympus IX71 microscope and the Cell F software (Olympus, Tokyo, Japan).

### Immunofluorescence for pluripotency markers

Detection of pluripotency markers in iPSCs was assessed by immunocytochemistry as previously described [8]. Images were acquired using a High Content screening system (HCS) ImageXpress XL (Molecular Devices, San Jose, California, U.S.). The software used to operate the HCS system and to analyze the images was the MetaXpress (Molecular Devices).

### In vivo differentiation: teratoma formation assay

The in vivo differentiation assay was performed with modifications [15], after approval by the National Animal Care Committee (CONCEA), protocol n° 018/2014. Approximately 2 × 10^6^ cells were injected subcutaneously at the nuchal region of 8–16-weeks-old male and female NOD-Scid IL-2Rɣ null (NSG) mice. Visible tumors were developed after 7–12 weeks when mice were euthanized. The teratomas were fixed, processed and stained with H&E. Images were obtained in an Observer.A1 microscope using the ZEN 2.3 lite software (Zeiss, Jena, Germany).

### Karyotyping of iPSCs

Genomic stability of the iPSCs was assessed by G-banding [26]. Briefly, 2 × 10^6^ single iPSCs were plated in a geltrex coated plate in mTeSR1 medium supplemented with 10 μM Rock inhibitor. The following day, medium was replaced by fresh mTeSR1 supplemented with 0.1 μg/mL Colcemid (Gibco, Waltham, Massachusetts, U.S.) and incubated for 16 h to arrest cells in metaphase. Cells were harvested, exposed to pre-warmed hypotonic solution (0.075 M KCl, 25 mM HEPES, pH 7.4) and fixed in methanol: acetic acid (3:1). G-banding was performed by 0.5% tripsin/PBS treatment and staining in Wright’s dye. Twenty metaphases were captured from each iPSCs line to mount the karyotype. The karyotypes were analyzed in the Applied Spectral Imaging software, Carlsbad, California, U.S.

### Hematopoietic differentiation from iPSCs

The hematopoietic differentiation was performed through the embryoid bodies (EBs) method in serum free conditions, as previously reported [8], with slight modifications. Briefly, EBs were cultured in STEMdiff APEL 2 (STEMCELL Technologies) supplemented with 30 ng/mL VEGF and BMP4, 40 ng/mL SCF and 50 ng/mL Activin A during 4 days. At day 4, medium was replaced by fresh STEMdiff APEL 2 containing 300 ng/mL SCF and FLT3L, 10 ng/mL of IL-3 and IL-6, 50 ng/mL G-CSF and 25 ng/mL BMP4. EBs were kept at 37 °C, 5% CO_2_ for 16 days. Cytokines were purchased from Peprotech, São Paulo, Brazil.

### Immunophenotyping of HSPCs

EBs were collected at day 16 of hematopoietic differentiation and dissociated into individual cells with Accutase (Gibco). For immunostaining, cells were incubated with anti-CD34 PE, anti-CD45 FITC and anti-CD43 APC-H7 (BD Biosciences). Viability was assessed with LIVE/DEAD fixable violet dead cell stain kit (Themo Fisher Scientifics) or 7-AAD (BD Biosciences). Cells were acquired for analysis using a LSRFortessa™ cytometer or FACSCanto™ II (BD Biosciences). Analysis was performed with FlowJo V10 (BD Biosciences).

### Colony-forming unit assay (CFU)

For the CFU assay, individualized cells from EBs at day 16 of hematopoietic differentiation were homogenized in MethoCult H4435 (STEMCELL Technologies) at a total number of 5 × 10^4^ (triplicates) and incubated at 37 °C, 5% CO_2_ for 14 days. Colonies were classified and quantified according to their morphology using an Olympus IX71 microscope and images were acquired with the Cell F software (Olympus).

### Real time quantitative PCR for telomere biology genes

RNA from iPSCs was isolated using TRIzol™ LS Reagent (Thermo Fisher Scientific). Then, the RNA was transcribed to cDNA using the High-Capacity cDNA Reverse Transcription kit (Thermo Fisher Scientific), as indicated by manufacturer. The RT-qPCR was performed with TaqMan^®^ Universal PCR Master Mix according to the manufacturer’s recommendations and the TaqMan™Gene Expression Assay (FAM) probes: *TERT*, *TERC*, *TINF2*, *POT1,* and *TRF2* (Thermo Fisher Scientific). The q-RT-PCR was performed in an Applied Biosystems 7500 Real-Time PCR system (Thermo Fisher Scientific). ESCs (H1 cell line) were the reference group. Fold changes were calculated using the ddCT method, in which fold change data were represented as 2^−ddCT^ [27]. Results were normalized to *GAPDH* expression.

### Telomerase activity assay

In vitro telomerase activity was assessed by TRAP (Telomeric repeat amplification protocol) assay using the TRAPeze XL Telomerase Detection kit (Millipore, Burlington, Massachusetts, U.S.), as previously described [8]. Telomerase activity of HeLa cells was considered as 100%. Telomerase activity of healthy and AA-iPSCs was represented as mean ± SD of three independent assays.

### Quantitative PCR for telomere length

Total DNA was extracted using the DNeasy Blood and Tissue kit (Qiagen, Hilden, Germany) following the manufacturer’s recommendations. The integrity of DNA was assessed by electrophoresis in agarose gel. The quantitative PCR for telomere length was performed as previously described [28].

### Data analysis

The quantitative analysis was based on at least three replicates. Data are presented as means ± SD and considered a confidence interval of 95%. Mann Whitney test for independent observations was performed using the statistic software GraphPad Prism version 5 (GraphPad).

## Supplementary information

Agreement from all authors with new author.

Suppl Figure legend

Supplementary Figures

Supplementary Table 1

Supplementary Method

Reproducibility checklist

## Data Availability

The data used to support the findings of this study are available from the corresponding author upon request.
