# Supplementary material for: Defective hematopoietic differentiation of immune aplastic anemia patient-derived iPSCs
Source: Cell Death Dis. 2022 Apr 28;13(4):412. doi: 10.1038/s41419-022-04850-5 (PMC9051057; doi:10.1038/s41419-022-04850-5)
Supplement: Supplementary file 1 — Agreement from all authors with new author. [file 41419_2022_4850_MOESM1_ESM.pdf]

Rodrigo do Tocantins Calado de Saloma Rodrigues <rtcalado@fmrp.usp.br>

Author addition to our manuscript to Cell Death & Disease

12 mensagens

Rodrigo T. Calado <rtcalado@fmrp.usp.br> 4 de abril de 2022 15:58
Para: florenciatell <florenciatell@usp.br>, Flávia Sacilotto Donaires <flaviadonaires@gmail.com>, Vinícius Silva de Carvalho <viniciussc@usp.br>, Barbara Amélia Aparecida Santana <barbarasantana@fmrp.usp.br>, FERNANDA BORGES DA SILVA <fernanda.borges@hemocentro.fmrp.usp.br>, raissatristao@usp.br, lilianfigueiredo <lilianfigueiredo@yahoo.com.br>, yordanka.yoya@gmail.com, Ligia Pereira <lpereira@usp.br>

Dear Co-Authors:

I am writing to you because we have added Dr Aline de Souza, from FZEA-USP, as a co-author to the manuscript, given that she significantly contributed with new cell lines to respond to the reviewer comments.
Having said that, I would like to receive your reply confirming that you agree with Dr. de Souza being a co-author in the manuscript, as requested by the Journal for final review.

Thank you for your attention to this matter,

Rodrigo T. Calado, MD/PhD
Chair, Department of Medical Imaging, Hematology, and Clinical Oncology
Ribeirão Preto School of Medicine
University of São Paulo

Av. Bandeirantes, 3900
Ribeirão Preto, SP 14049-900, Brasil
Tel.: +(55)(16) 3602-2037
E-mail: rtcalado@usp.br

Lygia da Veiga Pereira <lpereira@usp.br> 4 de abril de 2022 15:59
Para: rtcalado@fmrp.usp.br

Sua mensagem Para: Lygia da Veiga Pereira Assunto: Author addition to our manuscript to Cell Death & Disease Enviada em: 04/04/2022 15:58:29 BRT foi lida em 04/04/2022 15:59:42 BRT

Lygia da Veiga Pereira <lpereira@usp.br> 4 de abril de 2022 15:59
Para: "Rodrigo T. Calado" <rtcalado@fmrp.usp.br>

Confirmed!
[Texto das mensagens anteriores oculto]
--
Lygia V. Pereira, Ph.D.
Laboratório Nacional de Células-Tronco Embrionárias - USP
Depto. Genética e Biologia Evolutiva
Instituto de Biociências, USP
Rua do Matão, 277 sala 300
São Paulo, SP
Brasil

05508-090
tel. (55-11)3091-7476
fax.(55-11)3091-7553
skype: lygiavp

Yordanka Yoya <yordanka.yoya@gmail.com> 4 de abril de 2022 17:04
Para: "Rodrigo T. Calado" <rtcalado@fmrp.usp.br>
Cc: florenciatell <florenciatell@usp.br>, Flávia Sacilotto Donaires <flaviadonaires@gmail.com>, Vinícius Silva de Carvalho <viniciussc@usp.br>, Barbara Amélia Aparecida Santana <barbarasantana@fmrp.usp.br>, FERNANDA BORGES DA SILVA <fernanda.borges@hemocentro.fmrp.usp.br>, raissatristao@usp.br, lilianfigueiredo <lilianfigueiredo@yahoo.com.br>, Ligia Pereira <lpereira@usp.br>

Dear Dr Ricardo,
I agree with Dr de Souza being a co-author in the manuscript "Defective hematopoietic differentiation of immune aplastic anemia patient-derived iPSCs", as requested by the jornal Cell Death & Disease, for final review.

Sincerely,
Yordanka.

Em seg., 4 de abr. de 2022 às 15:57, Rodrigo T. Calado <rtcalado@fmrp.usp.br> escreveu:
[Texto das mensagens anteriores oculto]

--

Yordanka Medina Armenteros

Laboratório Nacional de Células-Tronco Embrionárias (LaNCE) – SP

Depto. Genética e Biologia Evolutiva

Universidade de São Paulo

Rua do Matão, 277, sala 300

São Paulo, SP

05508-090

Tel: +55 (11) 26488393
+55 (11) 30917476

---

lilian figueiredo <lilianfigueiredo@yahoo.com.br> 4 de abril de 2022 22:53
Para: Yordanka Yoya <yordanka.yoya@gmail.com>
Cc: "Rodrigo T. Calado" <rtcalado@fmrp.usp.br>, florenciatell <florenciatell@usp.br>, Flávia Sacilotto Donaires <flaviadonaires@gmail.com>, Vinícius Silva de Carvalho <viniciussc@usp.br>, Barbara Amélia Aparecida Santana <barbarasantana@fmrp.usp.br>, FERNANDA BORGES DA SILVA <fernanda.borges@hemocentro.fmrp.usp.br>, raissatristao@usp.br, Ligia Pereira <lpereira@usp.br>

Dear Dr Calado,

I agree with Dr Aline de Souza being a co-author in the manuscript "Defective hematopoietic differentiation of immune aplastic anemia patient-derived iPSCs", as requested by the jornal Cell Death & Disease, for final review.

Sincerely,
Lílian Figueiredo Moreira

Em 4 de abr. de 2022, à(s) 17:04, Yordanka Yoya <yordanka.yoya@gmail.com> escreveu:

[Texto das mensagens anteriores oculto]

Bárbara Amélia Aparecida Santana <barbarasantana@fmrp.usp.br> 5 de abril de 2022 07:41
Para: "Rodrigo T. Calado" <rtcalado@fmrp.usp.br>

sure

Bárbara Santana, PhD
Especialista em Laboratório
Hospital das Clínicas da FMRP-USP
Laboratório de Hematologia, Bloco G
Campus Universitário s/n
CEP: 14048-900 - Ribeirão Preto/SP

Em seg., 4 de abr. de 2022 às 15:57, Rodrigo T. Calado <rtcalado@fmrp.usp.br> escreveu:
[Texto das mensagens anteriores oculto]

FERNANDA BORGES DA SILVA <fernanda.borges@hemocentro.fmrp.usp.br> 5 de abril de 2022 09:31
Para: "Rodrigo T. Calado" <rtcalado@fmrp.usp.br>

Dear Dr Calado,

I agree with Dr Aline de Souza being a co-author in the manuscript "Defective hematopoietic differentiation of immune aplastic anemia patient-derived iPSCs", as requested by the jornal Cell Death & Disease, for final review.

Sincerely,
Fernanda Borges da Silva

Em seg., 4 de abr. de 2022 às 15:57, Rodrigo T. Calado <rtcalado@fmrp.usp.br> escreveu:
[Texto das mensagens anteriores oculto]

Aviso de Confidencialidade: Esta mensagem, incluindo seus anexos, tem caráter confidencial e seu conteúdo é restrito ao destinatário da mensagem. Se você não for o(a) destinatário(a) final ou recebeu esta mensagem por engano, você não pode copiar, utilizar, divulgar ou agir baseado nesta mensagem ou qualquer informação nela contida. Por gentileza comunique imediatamente o remetente desta mensagem e a remova de seu sistema. Qualquer uso não autorizado, replicação ou disseminação desta mensagem ou parte dela é expressamente proibido. As informações ou opiniões pessoais do remetente nela contidas podem não refletir o ponto de vista do Hemocentro RP e da FUNDHERP que apenas é divulgado por colaboradores devidamente autorizados. Eventuais Dados Pessoais contidos nesta mensagem, estão protegidos e em conformidade com a Lei Geral de Proteção de Dados - LGPD.

Maria Florencia Tellechea <florenciatell@usp.br> 5 de abril de 2022 09:51
Para: "Rodrigo T. Calado" <rtcalado@fmrp.usp.br>

Dear Dr Calado,

I agree with Dr Aline de Souza being a co-author in the manuscript "Defective hematopoietic differentiation of immune aplastic anemia patient-derived iPSCs", as requested by the jornal Cell Death & Disease, for final review.

Sincerely,
Maria Florencia Tellechea

Maria Florencia Tellechea, PhD
Faculdade de Medicina de Ribeirão Preto - Universidade de São Paulo
Laboratório de Hematologia - Hospital das Clínicas
Telefone: (16) 3602-2859

[Texto das mensagens anteriores oculto]

Vinícius Silva de Carvalho <viniciussc@usp.br> 5 de abril de 2022 12:08
Para: rtcalado@fmrp.usp.br

Sua mensagem Para: Vinícius Silva de Carvalho Assunto: Author addition to our manuscript to Cell Death & Disease
Enviada em: 04/04/2022 15:58:29 BRT foi lida em 05/04/2022 12:08:46 BRT

Vinícius Silva de Carvalho <viniciussc@usp.br> 5 de abril de 2022 12:08
Para: "Rodrigo T. Calado" <rtcalado@fmrp.usp.br>

Dear Dr Calado,

I agree with Dr de Souza being a co-author in the manuscript "Defective hematopoietic differentiation of immune aplastic anemia patient-derived iPSCs", as requested by the journal Cell Death & Disease, for final review.

Sincerely,
Vinícius Carvalho
[Texto das mensagens anteriores oculto]

Raíssa Silva Tristão <raissatristao@usp.br> 6 de abril de 2022 11:28
Para: lilian figueiredo <lilianfigueiredo@yahoo.com.br>
Cc: Yordanka Yoya <yordanka.yoya@gmail.com>, "Rodrigo T. Calado" <rtcalado@fmrp.usp.br>, florenciatell <florenciatell@usp.br>, Flávia Sacilotto Donaires <flaviadonaires@gmail.com>, Vinícius Silva de Carvalho <viniciussc@usp.br>, Barbara Amélia Aparecida Santana <barbarasantana@fmrp.usp.br>, FERNANDA BORGES DA SILVA <fernanda.borges@hemocentro.fmrp.usp.br>, Ligia Pereira <lpereira@usp.br>

Dear Dr Calado,

I agree with Dr Aline de Souza being a co-author in the manuscript "Defective hematopoietic differentiation of immune aplastic anemia patient-derived iPSCs", as requested by the jornal Cell Death & Disease, for final review.

Sincerely,
Raíssa S.Tristão
[Texto das mensagens anteriores oculto]

Flávia Sacilotto Donaires <flaviadonaires@gmail.com> 6 de abril de 2022 15:39
Para: Raíssa Silva Tristão <raissatristao@usp.br>
Cc: lilian figueiredo <lilianfigueiredo@yahoo.com.br>, Yordanka Yoya <yordanka.yoya@gmail.com>, "Rodrigo T. Calado" <rtcalado@fmrp.usp.br>, florenciatell <florenciatell@usp.br>, Vinícius Silva de Carvalho <viniciussc@usp.br>, Barbara Amélia Aparecida Santana <barbarasantana@fmrp.usp.br>, FERNANDA BORGES DA SILVA <fernanda.borges@hemocentro.fmrp.usp.br>, Ligia Pereira <lpereira@usp.br>

Dear Dr Calado,

I agree with the addition of Dr Aline de Souza as a co-author in the manuscript "Defective hematopoietic differentiation of immune aplastic anemia patient-derived iPSCs", as requested by the jornal Cell Death & Disease, for final review.

Sincerely,
Flávia Donaires
[Texto das mensagens anteriores oculto]
