# Supplementary material for: Defective hematopoietic differentiation of immune aplastic anemia patient-derived iPSCs
Source: Cell Death Dis. 2022 Apr 28;13(4):412. doi: 10.1038/s41419-022-04850-5 (PMC9051057; doi:10.1038/s41419-022-04850-5)
Supplement: Supplementary file 2 — Suppl Figure legend [file 41419_2022_4850_MOESM2_ESM.docx]

**SUPPLEMENTARY FIGURE LEGEND**

**Supplementary Figure 1. Agarose gel for detection of *EBNA1* in iPSCs lines.** The *EBNA1* fragment (amplicon size: 326 bp) is absent in all iPSC lines, ESCs line (H1) and HeLa (negative controls). Reprogramming episomal plasmids used to derive iPSC lines Ctrl-1, AA-1 to AA-3 were pCXB-EBNA1, pCE-mp53DD, pCE-hOCT3/4, pCE-hUL and pCE-hSK. The plasmids for deriving iPSC line Ctrl-2 were MBX, MOS and MMK. These plasmids served as positive controls for *EBNA1* amplification. M: marker (1 kb ladder). NTC: negative control. p: passage.

**Supplementary Figure 2. Hematopoietic progenitors express similar levels of CD43 and CD45.** Analysis of flow cytometry data showing the immunophenotype of hematopoietic progenitors derived from a control iPSC at day 16 of differentiation. After exclusion of doublets and dead cells, the CD43 marker was assessed on the CD34^+^CD45^+^ fraction. In our differentiation protocol, we observed that 97% of CD34^+^CD43^+^ cells also co-express the hematopoietic marker CD45. Sample corresponds to hematopoietic differentiation from Ctrl-1, clone 2 (2 independent experiments).

**Supplementary Figure 3. AA and healthy-iPSCs differentiation into hematopoietic progenitors.** Classification and quantification of hematopoietic colonies derived from healthy (3 subjects, 5 clones) and AA-iPSC (3 subjects, 5 clones). Data is presented as mean of experimental triplicates ± SD.
