# Supplementary figures and images for: Defective hematopoietic differentiation of immune aplastic anemia patient-derived iPSCs
Source: Cell Death Dis. 2022 Apr 28;13(4):412. doi: 10.1038/s41419-022-04850-5 (PMC9051057; doi:10.1038/s41419-022-04850-5)

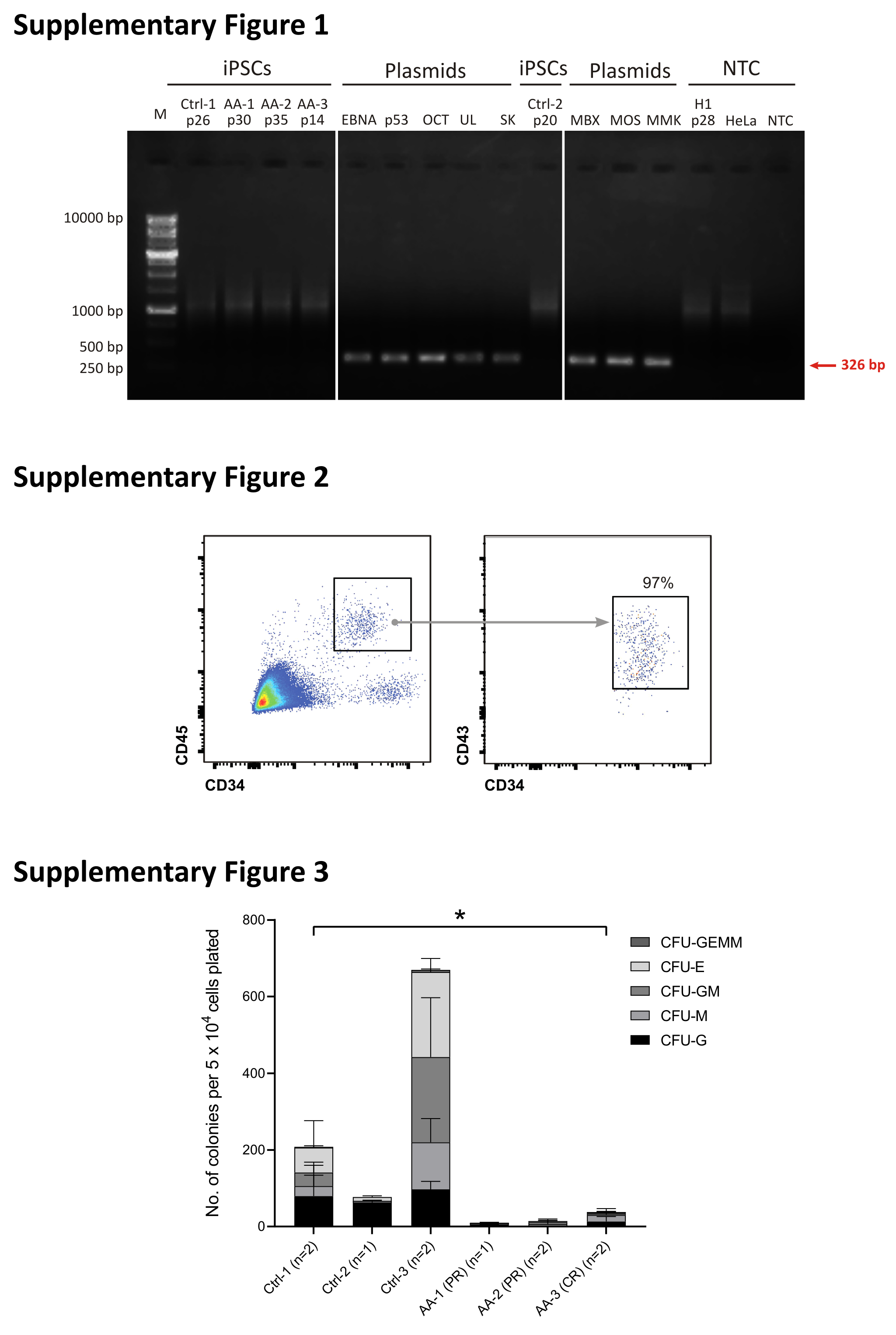

Supplement: Supplementary file 3 — Supplementary Figures [file 41419_2022_4850_MOESM3_ESM.tif]
