# Supplementary material for: Defective hematopoietic differentiation of immune aplastic anemia patient-derived iPSCs
Source: Cell Death Dis. 2022 Apr 28;13(4):412. doi: 10.1038/s41419-022-04850-5 (PMC9051057; doi:10.1038/s41419-022-04850-5)
Supplement: Supplementary file 4 — Supplementary Table 1 [file 41419_2022_4850_MOESM4_ESM.docx]

**Supplementary Table 1.** Panel of bone marrow failure genes.

| *ABCA3* | *CUX1* | *GFI1* | *NPM1* | *RPS26* | *TERF2* |
| --- | --- | --- | --- | --- | --- |
| *ABL1* | *DCLRE1B* | *GNAS* | *NRAS* | *RPS7* | *TERF2IP* |
| *ASXL1* | *DDX11* | *GRHL2* | *OBFC1* | *RTEL1* | *TERT*gene |
| *ATM* | *DDX41* | *HAX1* | *PALB2* | *RUNX1* | *TERT*promoter |
| *ATRX* | *DHX36* | *HRAS* | *PARN* | *RUVBL1* | *TERT*rs2736100 |
| *BCOR* | *DIDO1* | *IDH1* | *PCNA* | *RUVBL2* | *TET2* |
| *BCORL1* | *DKC1* | *IDH2* | *PDGFRA* | *SAMD9L* | *TGS1* |
| *BLM* | *DNMT3A* | *IKZF1* | *PEG3* | *SBDS* | *THPO* |
| *BRAF* | *ELANE* | *IRF1* | *PHF6* | *SETBP1* | *TINF2* |
| *BRCA2* | *ETV6* | *JAK1* | *PIF1* | *SF3B1* | *TOLLIP*rs111521887 |
| *BRIP1* | *EZH1* | *JAK2* | *PIGA* | *SFTPA1* | *TOLLIP*rs5743890 |
| *CALR* | *EZH2* | *JAK3* | *PML* | *SFTPA2* | *TOLLIP*rs5743894 |
| *CBL* | *FANCA* | *KDM6A* | *POT1* | *SFTPB* | *TP53* |
| *CBLB* | *FANCB* | *KIT* | *PRF1* | *SFTPC* | *TPP1* |
| *CBLC* | *FANCC* | *KMT2A* | *PTEN* | *SH2B3* | *U2AF1* |
| *CCT2* | *FANCD2* | *KRAS* | *PTGES3* | *SHQ1* | *U2AF2* |
| *CCT3* | *FANCE* | *LIG4* | *PTPN11* | *SLX4* | *USB1* |
| *CCT4* | *FANCF* | *MPL* | *RAD21* | *SMC1A* | *WAS* |
| *CCT5* | *FANCG* | *MRE11A* | *RAD51C* | *SMC3* | *WRAP53* |
| *CCT6A* | *FANCI* | *MUC5B* | *RPL11* | *SRP72* | *WRN* |
| *CCT7* | *FANCL* | *MYC* | *RPL15* | *SRSF2* | *WT1* |
| *CCT8* | *FANCM* | *MYD88* | *RPL35A* | *STAG2* | *ZBTB48* |
| *CDAN1* | *FBXW7* | *NAF1* | *RPL5* | *STAT3* | *ZRSR2* |
| *CDKN2A* | *FLT3* | *NBN* | *RPS10* | *TCP1* |  |
| *CEBPA* | *G6PC3* | *NHP2* | *RPS17* | *TEN1* |  |
| *CSF3R* | *GATA1* | *NOP10* | *RPS19* | *TERC* |  |
| *CTC1* | *GATA2* | *NOTCH1* | *RPS24* | *TERF1* |  |
