# Supplementary material for: Defective hematopoietic differentiation of immune aplastic anemia patient-derived iPSCs
Source: Cell Death Dis. 2022 Apr 28;13(4):412. doi: 10.1038/s41419-022-04850-5 (PMC9051057; doi:10.1038/s41419-022-04850-5)
Supplement: Supplementary file 5 — Supplementary Method [file 41419_2022_4850_MOESM5_ESM.docx]

**Supplementary method**

**Verification of plasmid integration in iPSCs**

To assess for reprogramming vector integration in the iPSC lines, we performed a PCR assay to amplify a region of the Epstein–Barr nuclear antigen 1 (*EBNA1*) which is common to the 8 episomal plasmids used to reprogram the cells for this study. First, we extracted genomic DNA from iPSCs at different passages (14-35) using the DNeasy Blood and Tissue kit (Qiagen), according to the manufacturer's recommendations. The PCR was performed using the HotStarTaq DNA Polymerase (Qiagen) and 10 ng of DNA per reaction. Plasmids were used as positive controls and the cell lineages H1 and HeLa, as negative controls. Primer sequences: EBNA_Fw: 5’ GGGTAGAGGACGTGAAAGAG 3’ and EBNA_Rv: 5’ CTCCTTTTTTGCGCCTGCCT 3’. Amplicon size: 326 bp.
